# Plasmonic Coaxial Waveguides with Complex Shapes of Cross-Sections

**DOI:** 10.3390/ma4010104

**Published:** 2010-12-31

**Authors:** Olga Kozina, Igor Nefedov, Leonid Melnikov, Antti Karilainen

**Affiliations:** 1 Saratov Branch of the Institute of Radio-Engineering and Electronics of Russian Academy of Science/Zelyonaya 38, Saratov 410019, Russia; E-Mail: kozinaolga@yandex.ru; 2 SMARAD Center of Excellence, Department of Radio Science and Engineering, School of Science and Technology, Aalto University, P.O. Box 13000, Aalto 00076, Finland; 3 Saratov State Technical University/Polytechnicheskaya 77, Saratov 410054, Russia; E-Mail: lam-pels@yandex.ru

**Keywords:** nano-size, plasmonic waveguides, coaxial

## Abstract

In this paper, we describe waveguide properties of new optical waveguides made of noble metals and filled with glass and air. Such waveguides are coaxial cables and differ from a conventional coaxial in the shape of their central rods. Coaxial waveguide with annular and elliptic central rods are considered. Numerical simulations demonstrate that these waveguides, having nanosize cross-section, support propagation of few comparatively low-loss modes, having phase velocity close to the speed of light and the fields localized in a small area outside a metal. We illustrate excitation of these coaxial modes by dipole-like sources.

## 1. Introduction

Miniaturization of optical components is now a topical challenge and goes a long way towards nanotechnologies. Artificial optical systems comprised of subwavelength elements (waveguides, resonators, *etc*.) have recently been proposed, studied, and engineered. Here, subwavelength sizes refer only to waveguide cross-sections (*i.e.*, not to the longitudinal direction), which should be much smaller than the half-wavelength, *λ/*2. In this way, the goal is to create optical nano-size waveguides to support the propagation of light to a needed distance with small losses. As known, there are various kinds of photonic crystal fibers, including fibers with subwavelength channels, for transmission of radiation in the optical range. However, electromagnetic field localization is limited by few microns in these fibers.

One of the promising ways to miniaturize optical waveguides is to use surface plasmon-polaritons propagating in noble metals. However, light propagation length is very short in plasmonic waveguides as compared with optical fibers. Thus, a very important task is to reduce absorption losses in waveguides, keeping their nanoscale sizes. Different types of plasmonic waveguides have been studied: metal films of different width [1,2], hollow metal waveguides [3], metal cylinders with and without dielectric core [4,5], metal wedges [6], and coaxial waveguides [7,8,9,10,11,12]. The numerical method, based on Green’s tensor, for calculation of plasmon properties of single-hole and pair-hole structures in thin gold films was developed in [10]. For some applications, closed waveguides are preferable since they provide confinement of light inside the waveguide cross section. At the same time, reduction of the cross section usually causes an increase of attenuation. It was found in [8] that some modes in metal coaxial waveguides exhibit considerably lower losses compared with hollow plasmon waveguides at optical frequencies. Fields of these modes are the result of interaction of plasmons and electromagnetic waves in the air region. The real part of the effective refractive index for modes possessing the lowest attenuation lies between 1 and 2.

It is an attractive idea to use coaxial cables similar to those applied at radio frequencies. In such waveguides, made of well-conductive metals in radio and microwave technique, the TEM mode can propagate without cutoff. It was found that coaxial waveguides made of noble metals also support propagation of the azimuthally-symmetric TEM-like plasmonic mode in the optical range [7,8,9]. Coaxial plasmon waveguides are of special interest due to smaller attenuation of the TEM-like mode compared with any mode in the same-size hollow plasmon waveguide [7,8]. This mode has a symmetric field distribution and has no cutoff [8] that gives ground to use the term “superenhanced light transmission” [11]. In opposite to the TEM mode in the coaxial made of perfect electric conductor (PEC), it has a nonzero axial component of the electric field. Dispersion in coaxial waveguides, made of silver, was experimentally investigated in the visible range in [12]. Enhanced transmission through two-dimensional subwavelength arrays of coaxial (or annular aperture) holes was discussed in a series of works [11,13,14,15,16]. Arrays of coaxial holes can be used in the optical range as frequency selective surfaces (FSS) [16] and volumes (FSV), for optical spatial filtering [19] and in other applications based on extraordinary transmission.

In this paper, we investigate coaxial waveguides in which the bulk inner metal rod is replaced by one or several thin metal annuli having elliptic shapes. We demonstrate that a strong field localization can be achieved in coaxial waveguides with a circular shaped annulus filled with the glass inside. The phase velocity of the dipole-like mode in this waveguide is close to the speed of light. We compare optical properties of such a waveguide with a conventional coaxial. Then, we discuss excitation of the dipole-type mode by a point-like source in coaxials with elliptic-type central rods. We also demonstrate that modes with two orthogonal polarizations can be excited in structures with two crossed elliptic rods.

## 2. Coaxial Waveguide with Annular Central Rods

In the optical range, the electromagnetic properties of metals are described by a complex dielectric constant. The real part is negative and the imaginary part takes losses into account. It is important to notice, that for noble metals (silver, gold), the losses remain small and the imaginary part of the dielectric constant can be much smaller than its real part. The propagation constant is directly related to the effective refractive index of the mode *β* = *n*_eff_*ω/c*, where *c* is speed of light. In general, *n*_eff_ is a function of the frequency *ω* and it depends upon the geometry and on the type of mode. In the lossless case for propagating modes *β* is real and it is purely imaginary for evanescent modes.

All simulations have been performed using COMSOL Multiphysics at the wavelength *λ* = 500 nm. This model used is a direct numerical solution of Maxwell equations with proper boundary conditions. Silver is used as the metal and glass with the refractive index *n_g_* = 1.5 is used as the dielectric. The Ccomplex refractive index of silver *n_s_* for this wavelength was taken from [10]: *n_s_* = 0.05 + *i*3.093, and the corresponding permittivity is: *ε_s_ = n^2^_s_ = −*9.546 *+ i*0.309. The distance of light propagation along the waveguide is determined by *n*_eff_” *=* Im(*n*_eff_) and the estimated propagation length can be calculated as *L* *=* *λ*/(4*πn*_eff_”).

### 2.1. The Coaxial Waveguide with Two Nano-Sized Circular Tubes

**Figure 1:**
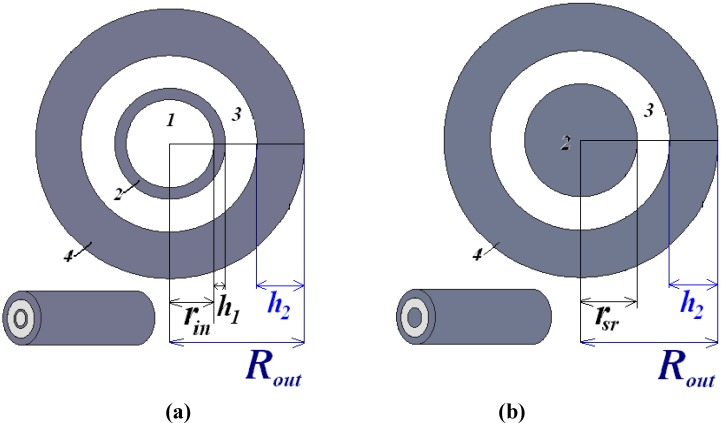
Schematic view of the coaxial waveguide with circular shape. (**a**) The coaxial waveguide consisting of two nano-sized metal tubes (annuli in cross sections; grey areas *2* and *4*) with a dielectric material between them (white area *3*) and an inner road filled with the same dielectric (white area *1*); (**b**) The coaxial waveguide with bulk inner rod (grey area *2*).

We first consider a coaxial waveguide consisting of two nano-sized metal tubes (which look as annuli in cross sections) with a dielectric material between them and an inner road filled with the same dielectric or bulk inner rod, see Figure 1 (a).

We denote cross-section radii of coaxial waveguides as the following: the radius of internal (glass) rod is *r*_in_, the external radius of the metal shell is *R*_out_, the thickness of the inner metal annulus is *h*_1_ and the thickness of the outer metal annulus is *h*_2_. The white areas denoted in Figure 1 (a) as *1* and *3* correspond to glass and the gray areas denoted as *2* and *4* correspond to metal.

We focus our attention on dipole-like modes as easily excited by dipoles. As known, these modes play a main role in extraordinary transmission through two-dimensional subwavelength arrays of coaxial holes [11,12,13,14,15,16,17,18].

**Figure 2:**
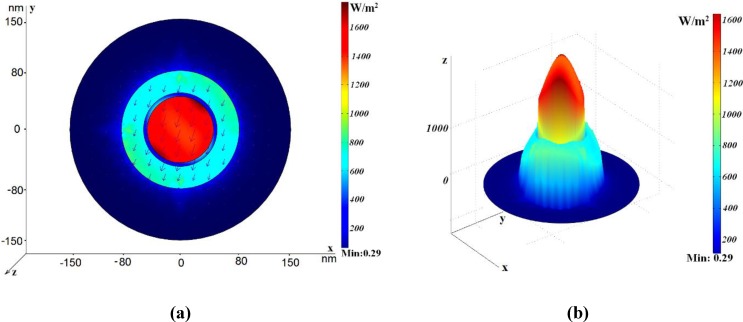

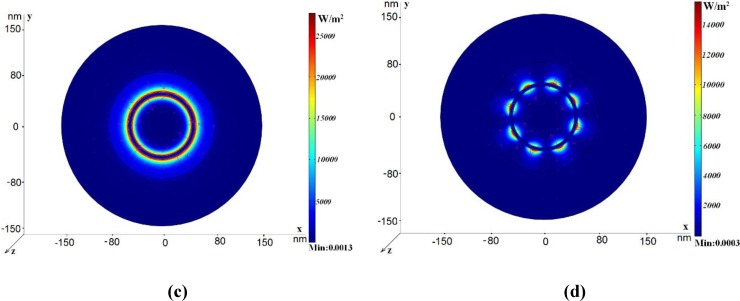
Power flow distribution for the various modes in the case *r*_in_ = 45 nm, *h*_1_ = 5 nm, *h*_2_ = 70 nm, *R*_out_ = 150 nm. The electric field vector is shown by arrows. (**a**) The dipole-like mode, *n*_eff_ = 0.742 + *i*0.026, *L* = 1.5 μm; (**b**) Three-dimensional power flow distribution for the same mode; (**c**) *n*_eff_ = 0.659 + *i*2.51, *L* = 0.015 μm. (**d**) *n*_eff_ = 4.32 + *i*0.42, *L* = 0.09 μm.

Let us consider the first kind of the coaxial with the inner road filled with the same dielectric (the central rod consisting of one tube). Parameters of this structure are the following: *r*_in_ = 45 nm, *h*_1_ = 5 nm, *h*_2_ = 70 nm, *R*_out_ = 150 nm. The radius *R*_out_ is chosen in such a way that the metallic outer boundary does not affect the propagation characteristics. In Figure 2, the power flow distribution for different modes is shown. In Figure 2 (a), the power flow distribution for the dipole-like mode (referred to in [8] as the TE_11_ mode, though it is a hybrid mode) is shown using artificial colors for intensity coding. Red color corresponds to the highest intensity. Arrows show the electric vector field. The effective refractive index of this mode is *n*_eff_ *=* 0.742 + *i*0.026, so its real part is considerably lower than the refractive index of glass. The electric field vector distribution corresponds to the dipole-like character of this mode. Figure 2 (b) illustrates the three-dimensional intensity distribution and demonstrates strong field localization in the central area of the waveguide. Figure 2 (c) demonstrates the azimuthally-symmetric plasmonic mode. The real part of its effective refractive index is less than unity, so its phase velocity is larger than the speed of light. However, it has high attenuation. Figure 2 (d) illustrates the electric field distribution for another slow non-symmetric plasmonic mode.

### 2.2. The Conventional Coaxial Waveguide with Nano-Sized Circular Rod

**Figure 3:**
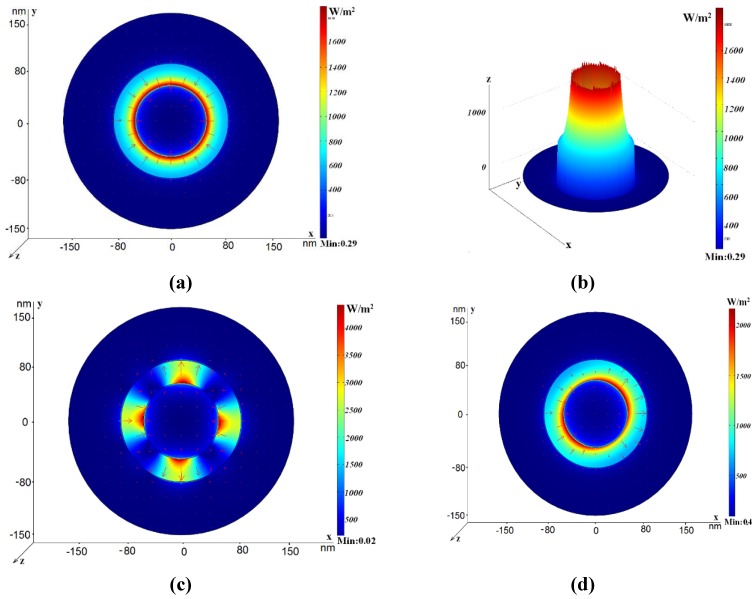
Power flow distribution for the various modes in the case *r*_silver rod_ = 50 nm, *h*_2_ = 70 nm, *R*_out_ = 150 nm. The electric field vector is shown by arrows. (**a**) *n*_eff_ = 3.027+ *i*0.037, *L* = 1.32 μm; (**b**) Three-dimensional power flow distribution for the same waveguide; (**c**) *n*_eff_ = 1.33 + *i*0.057, *L* = 0.69 μm; (**d**) *n*_eff_ = 2.61 + *i*0.36, *L* = 1.1 μm.

We compare the optical properties of such a waveguide with the conventional coaxial waveguide, see Figure 1 (b). For comparison, power flow distribution in a conventional coaxial waveguide with a bulk inner rod is shown in Figure 3 for three different modes. Figure 3 (a) corresponds to the mode with the effective refractive index *n*_eff_ *=* 3.027 + *i*0.037. The electric field vector distribution is similar to the TEM-like mode. Figure 2 (b) illustrates the three-dimensional intensity distribution for this mode. In Figure 3 (c), the quadrupole-like mode with the effective refractive index *n*_eff_ *=* 1.33 + *i*0.057 is shown. This is a fast mode, since its real part is lower than the refractive index of glass. Figure 3 (d) demonstrates the mode with the effective refractive index *n*_eff_ *=* 2.61 + *i*0.36. One can see that the real part is much larger than the refractive index of glass, such that it is slow wave, and this mode looks as the dipole-like mode. All three modes have considerably higher losses than the dipole-like mode in the coaxial with the annular cross-section, see discussion of Figure 2.

### 2.3. The Coaxial Waveguide with Multiple Nano-Sized Circular Annuli

**Figure 4:**
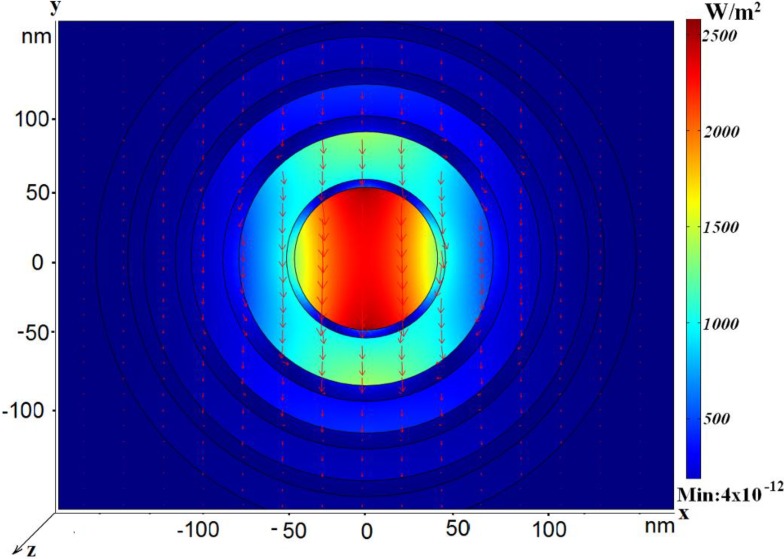
Power flow distribution for the dipole-like mode in case *r*_in_ = 45 nm; *h*_silver1_ = 5 nm; *h*_glassr1_ = 30 nm; *h*_silver2,3,4_ = 10 nm; *h*_glass2,3,4_ = 20 nm; *R*_out_ → ∞; *L* = 1.67 μm, *n*_eff_ = 1.010 + *i*0.0245. The electric field vector is shown by arrows.

Next we consider another type of the coaxial nanosize waveguide. We propose to surround the central part with periodically arranged aligned tubes. Appropriate choice of the geometry allows minimization of the absorption. The model of the coaxial waveguide, consisting of many nano-size metal tubes, is shown in Figure 4.

**Figure 5:**
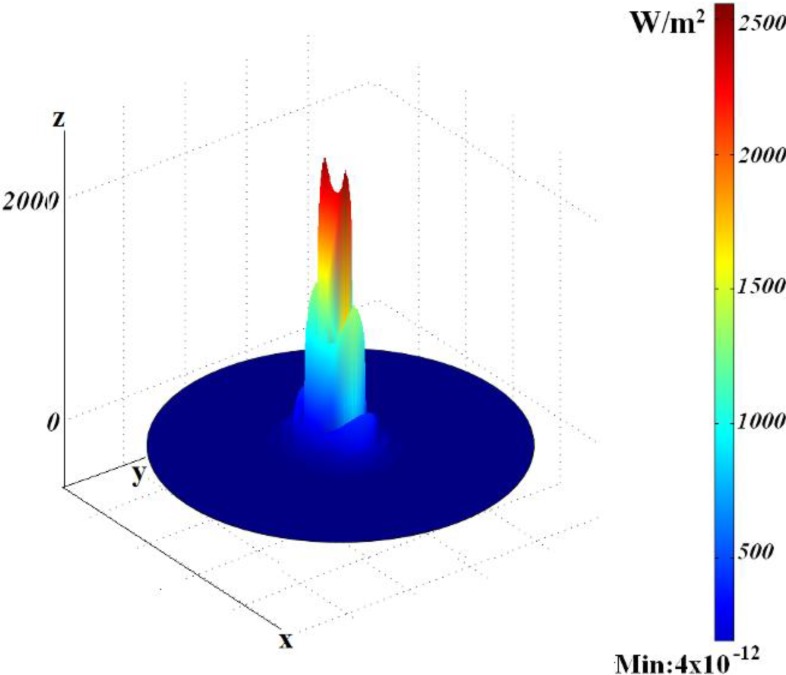
Three-dimensional power flow distribution.

In this case, if annuli are cut periodically in the metal outer shell, the metal part in the structure can be reduced. This allows stronger field localization in the inner dielectric part and small attenuation for fast modes (propagating with the phase velocity exceeding the speed of light in the glass). An appropriate choice of cuts makes it possible to improve confinement of the field in the central area, see Figure 5.

## 3. Coaxials with Elliptic-Type Central Rods

### 3.1. Field Distribution for Eigenmodes

**Figure 6:**
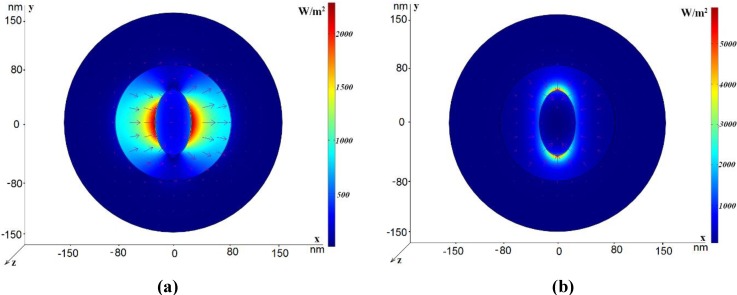
Power flow distribution for the dipole-like mode in the case *R*_out_ = 150 nm, *h*_2_ = 70 nm; the large ellipse semi-axis is 45 nm and the small ellipse semi-axis is 25 nm. The electric field vector is shown by arrows. (**a**) The dipole-like mode *n*_eff_ = 1.67 + *i*0.018; (**b**) The TEM-like mode *n*_eff_ = 3.06 + *i*0.046.

**Figure 7:**
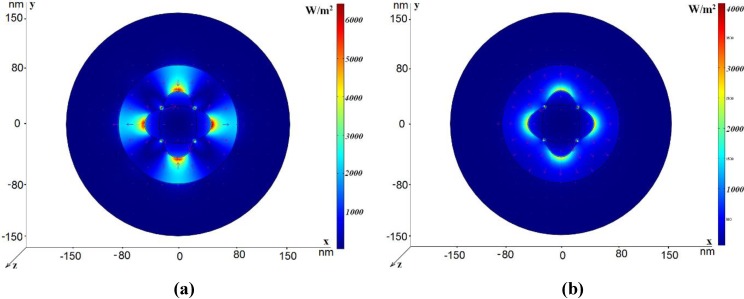
Power flow distribution for two modes in the case *R*_out_ = 150 nm, *h*_2_ = 70 nm; the large ellipse semi-axis is 45 nm and the small ellipse semi-axis is 25 nm. The electric field vector is shown by arrows. (**a**) The quadrupole-like mode, *n*_eff_ = 1.206 + *i*0.09; (**b**) The TEM-like mode, *n*_eff_ = 3.022 + *i*0.042.

In this section, we consider eigenmodes propagating in nano-cables with elliptic-type rods and rods with the shape of crossed ellipses. Figure 6 (a) and Figure 6 (b) illustrate the electric field distribution of the dipole-like mode and the TEM-like mode, respectively, in the coaxial with elliptic central rod. The filling medium is glass. The external radius of the structure is *R*_out_ = 150 nm and *h*_2_ = 70 nm, as before. The large ellipse semi-axis is 45 nm and the small ellipse semi-axis is 25 nm. The effective refractive index is *n*_eff_ = 1.67 + *i*0.018 for the dipole-like mode and *n*_eff_ = 3.06 + *i*0.046 for the TEM-like mode, so the dipole-like mode is faster and it possesses lower attenuation. Remember that the dipole-like mode, propagating in the coaxial with the circular central rod, having a radius 45 nm, is characterized by the effective refractive index *n*_eff_ = 2.61 + *i*0.36. Thus, modification of the central rod shape significantly changes both the real and imaginary parts of the effective refractive index for the dipole-like mode. The similar changes are much smaller for the TEM-like mode: (*n*_eff_ = 3.027 + *i*0.037 for the circular rod and *n*_eff_ = 3.06 + *i*0.046 for the ellipse). Earlier we proposed a coaxial with the central rode with the shape of two crossed ellipses [21]. Typical electric field distribution in such a coaxial is shown in Figure 7 (a) and Figure 7(b). The use of two crossed ellipses allows one to transmit independently two orthogonal polarizations with equal values of the effective refractive index (*n*_eff_ = 1.206 + *i*0.09). Each of these modes can be excited by an electric dipole (of the orthogonal polarization) and such waveguides can be used to transfer a dual-polarized field distribution, creating a dual-polarized image with a sub-wavelength resolution at a distance from the source. This structure also supports propagation of the TEM-like mode with the refractive index *n*_eff_ = 3.022 + *i*0.042. We pay special attention to the dipole-like modes because they can be easier excited by electric dipoles.

**Figure 8:**
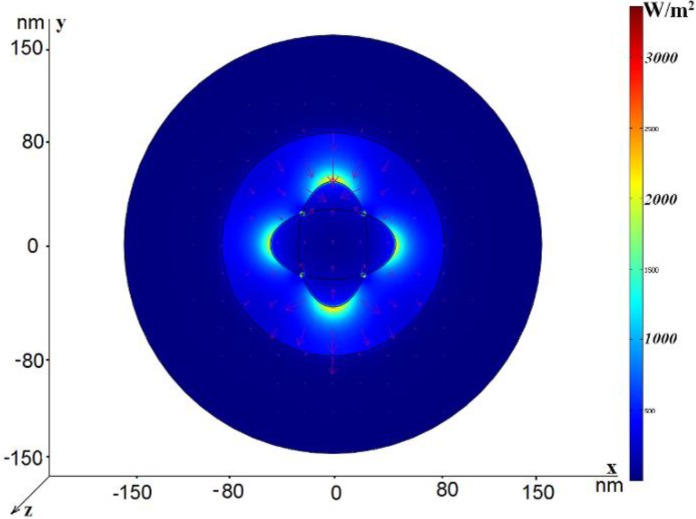
Power flow distribution for the dipole-like mode in the case R_out_ = 150 nm, h_2_ = 80 nm; the large ellipse semi-axis is 45 nm and the small ellipse semi-axis is 25 nm. n_eff_ = 2.396 + i0.045.

Electric field distribution for the dipole-like mode is shown in Figure 8. The imaginary part of the effective refractive index is larger than for the similar mode in the coaxial with the elliptic central rod, but is practically the same as for the TEM-like mode both in the coaxials with one ellipse and crossed ellipses.

### 3.2. Excitation by a Dipole

In this subsection, we consider excitation of modes in a finite-length coaxial by an elementary electric dipole placed near an edge of the coaxial. Waveguide simulations were done with Ansoft’s HFSS 11. The material parameters for silver are the same as used in previous simulations. The length of the coaxial segment is taken to be 500 nm and equals to the wavelength in free space. The dipole is placed at a distance of 20 nm from the coaxial edge.

**Figure 9:**
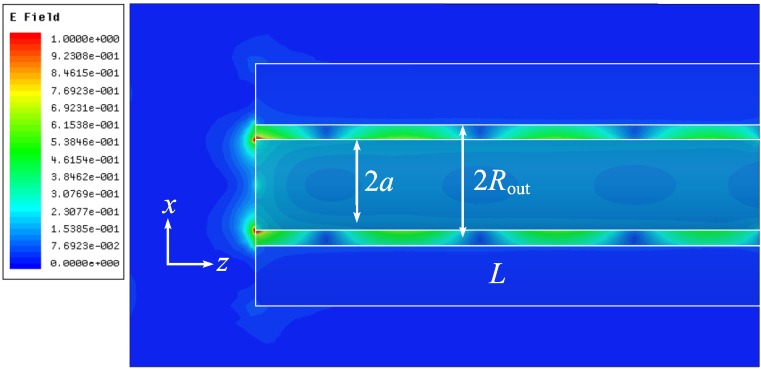
|*E(x,y,z)*|, phase-dependent modulus of the electric field. The large ellipse semi-axis *a* = 25 nm, the small semi-axis *b* = 45 nm, and *R*_out_ = 60 nm.

Figure 9 shows the electric field distribution along the coaxial with the elliptic central rod (the dipole is directed along the large ellipse axis). This plot demonstrates rather small attenuation of the dipole-like mode at the distance of 500 nm that is the wavelength in free space and almost two wavelengths in the coaxial (wavelength in the coaxial equals 280 nm).

**Figure 10:**
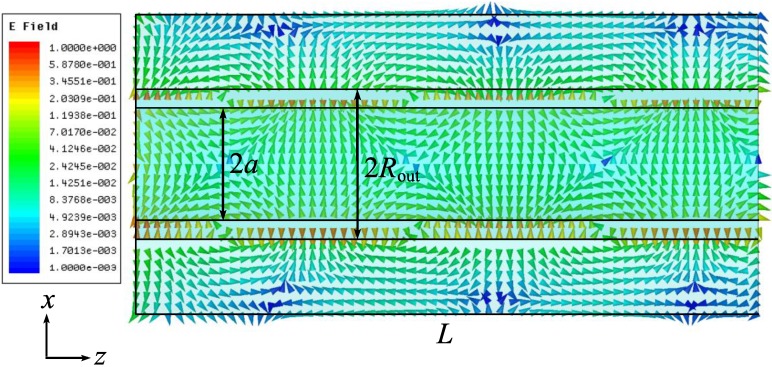
Re{*E(x,y,z)*}, phase dependent electric vector field in the *xz* plane.

**Figure 11:**
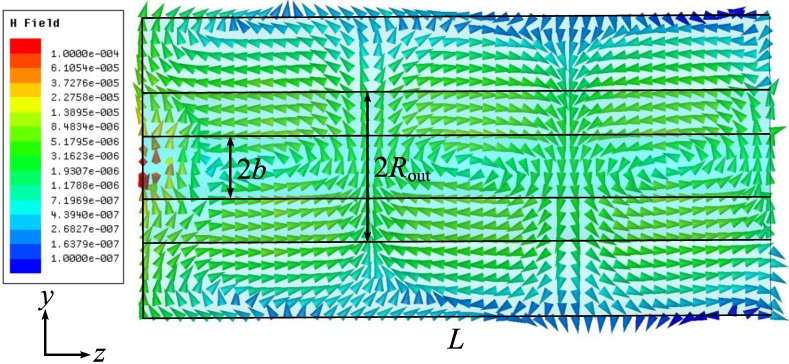
Re{*H(x,y,z)*}, phase dependent magnetic vector field in the *yz* plane.

Next we consider the field structure for the dipole-like mode. Figure 10 illustrates the electric vector field distribution along the coaxial in the *xz*-plane, the dipole plane (the dipole is directed along the large ellipse axis). The magnetic field vectors are orthogonal to the *xz*-plane and the magnetic vector field distribution is shown in the orthogonal, the *yz* plane, see Figure 11. The axial component of the magnetic field vector is nonzero in the *yz* plane, so it is more correct to refer to this mode as a hybrid mode, not as the TE_11_ (transversally-electric) mode, as in [8,15,18].

**Figure 12:**
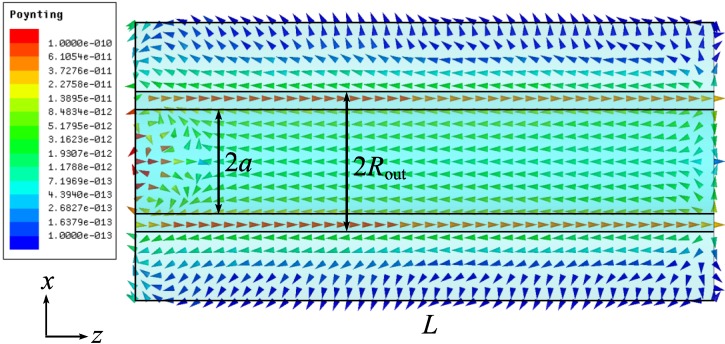
Time-averaged real part of the Poynting vector in the *xz* plane.

Figure 12 shows the Poynting vector distribution along the coaxial in the *xz* plane. It is seen that the energy flow propagates in the opposite directions in the air and metal areas. The most part of energy is transferred in the area close to the *xz* plane (the dipole plane).

**Figure 13:**
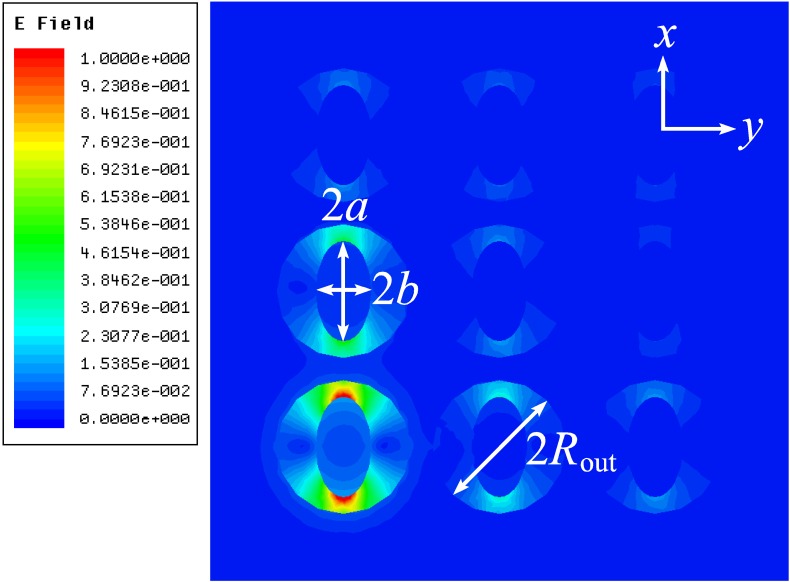
|*E(x,y,z)*|, the phase-dependent modulus of the electric field in an array of coaxials; *a* = 25 nm, *b* = 45 nm and *R*_out_ = 60 nm.

For demonstration of enhanced optical transmission through a periodically nanostructured metal slab, we consider excitation of an array of coaxial waveguides by the elementary electric dipole source. The array consists of nine coaxials in the silver matrix, as seen in Figure 13. The silver matrix is semi-infinite, starting at *z* = 0 nm. The waveguides in the matrix have the same inner dimensions as in Figure 7 (b). The dipole is located in air at the same distance of *z* = −20 nm from the interface as before, at the center of the axis of the bottom-left coaxial waveguide. Figure 13 shows the modulus of the complex electric field at *z* = 150 nm from the start of the waveguides. The energy spreads to the nearest neighboring waveguides, but not significantly.

## 4. Conclusions

In conclusion, we have investigated propagation and excitation of electromagnetic waves in coaxial waveguides, made of silver and having complex shaped central rods. We have demonstrated that strong subwavelength field localization can be achieved for the dipole-like mode in coaxial waveguides having glass filled annulus as the central rods. Other plasmonic-like modes have a considerably high attenuation factor. We have studied the coaxial waveguide with periodically cut annuli and have found that an appropriate choice of parameters allows the phase velocity to be close to the speed of light in free space, which can make excitation of the coaxial by an external dipole easier. We have discussed propagation of different eigenmodes in coaxial waveguides with elliptic-type central rods and have compared their attenuation. Distribution of the z-component of the Poynting vector demonstrates that energy flows have opposite directions in metal and air areas. Considered waveguide structures can be fabricated using the state-of-the-art techniques for producing photonic crystal glass. Complicated micro- and nanostructures manufacturing is possible via multiple redrawing and sintering of glass stacks [22,23,24,25]. Such nano-sized waveguides can be used, for example, as optical near-field probes and for transfer of images with subwavelength resolution.
